# Strategic Intervention: Ectopic Premolar Management for Preventing Molar-Premolar Transposition in Class III Malocclusion

**DOI:** 10.7759/cureus.58034

**Published:** 2024-04-11

**Authors:** Anjali Kathade, Rizwan Gilani, Shefali Singh

**Affiliations:** 1 Department of Orthodontics, Sharad Pawar Dental College, Wardha, IND

**Keywords:** maxillary arch abnormalities, multidisciplinary approach, orthodontic intervention, anterior crossbite, class iii malocclusion, ectopic premolar

## Abstract

Class III malocclusion often leads to the ectopic eruption of premolars in the upper arch, posing unique challenges for orthodontic practitioners. This case report explores the clinical implications of ectopic maxillary premolars in a Class III malocclusion patient, emphasizing the importance of early intervention and comprehensive treatment strategies. Factors contributing to ectopic eruption, including genetic predisposition and anatomical variations, are discussed, guiding orthodontists in effective diagnosis and management. A 14-year-old patient with Class III malocclusion, anterior crossbite, and ectopic maxillary premolars sought orthodontic consultation. The treatment plan involved extracting the deciduous second molar, realigning the ectopic premolar, and addressing arch length discrepancies. Utilizing fixed orthodontic appliances and strategic force application, the patient achieved Class I molar and canine relationships, resolving the ectopic premolar alignment within 10 months. Ectopic eruption of maxillary premolars, especially in Class III malocclusion, is a critical concern for oral health. Genetic predisposition, arch crowding, and developmental disturbances contribute to this condition. Early intervention, as demonstrated in this case, plays a pivotal role in restoring dentoskeletal harmony. The study underscores the need for a multidisciplinary approach, combining orthodontic, surgical, and restorative interventions for optimal outcomes. Thus, this case report highlights the successful management of ectopic maxillary premolars in a Class III malocclusion patient through strategic orthodontic intervention. Understanding the etiological factors and employing a comprehensive treatment approach facilitate timely diagnosis and prevent complications. Orthodontists must navigate the complexities of ectopic eruption, considering occlusal effects and collaborating with other specialists for holistic patient care.

## Introduction

The abnormal eruption of premolars in the upper arch, especially in individuals with a Class III malocclusion, draws in orthodontic practitioners with a unique array of challenges and factors to consider [1]. A Class III malocclusion, identified by the forward displacement of the lower teeth relative to the upper teeth, frequently impacts the eruption patterns of premolars, leading to their ectopic placement. This intersection of malocclusion and ectopic eruption necessitates a comprehensive understanding of the underlying factors and treatment approaches [2].

Understanding the factors contributing to ectopic eruptions is essential for early detection, diagnosis, and effective management of this condition. Factors such as genetic predisposition, local anatomical variations, and environmental influences may all contribute to the aberrant eruption patterns observed in premolars within the maxillary arch [3]. The incidence rate of impaction in premolars is roughly 0.5%. Mandibular premolars are noted to have a higher prevalence compared to maxillary ones. Mandibular second premolars are estimated to represent nearly 24% of all impactions, excluding molars [4]. This case report aims to provide an overview of the ectopic eruption of premolars in the maxillary arch in a Class III malocclusion patient, shedding light on its clinical implications, potential complications, and the importance of timely intervention. As we explore the intricacies of this dental issue, further investigation will delve into diagnostic standards, methods of treatment, and the orthodontic factors crucial for achieving optimal dental well-being and functionality. As we explore the intricacies of this dental issue, further investigation will delve into diagnostic standards, methods of treatment, and the orthodontic factors crucial for achieving optimal dental well-being and functionality.

## Case presentation

**Figure 1:**
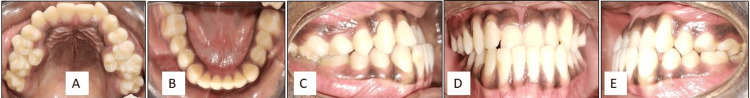
(A) Maxillary arch with ectopic premolar and over-retained second deciduous mola. (B) Mandibular arch. (C) Right buccal view. (D) Front view with negative overjet. (E) Left buccal view

**Figure 2:**
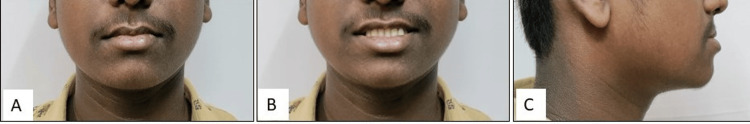
(A) Front view. (B) Smiling. (C) Profile view showing negative lip step

A 14-year-old patient sought consultation in the department due to concerns about the misplacement of the upper second premolar and an anterior crossbite. The intraoral assessment revealed an anterior crossbite and a Class III molar relationship (Figure 1). Upon extraoral examination, a skeletal Class III pattern with a low-angle case was noted, accompanied by reduced malar prominence and a negative lip step (Figure 2).

**Figure 3:**
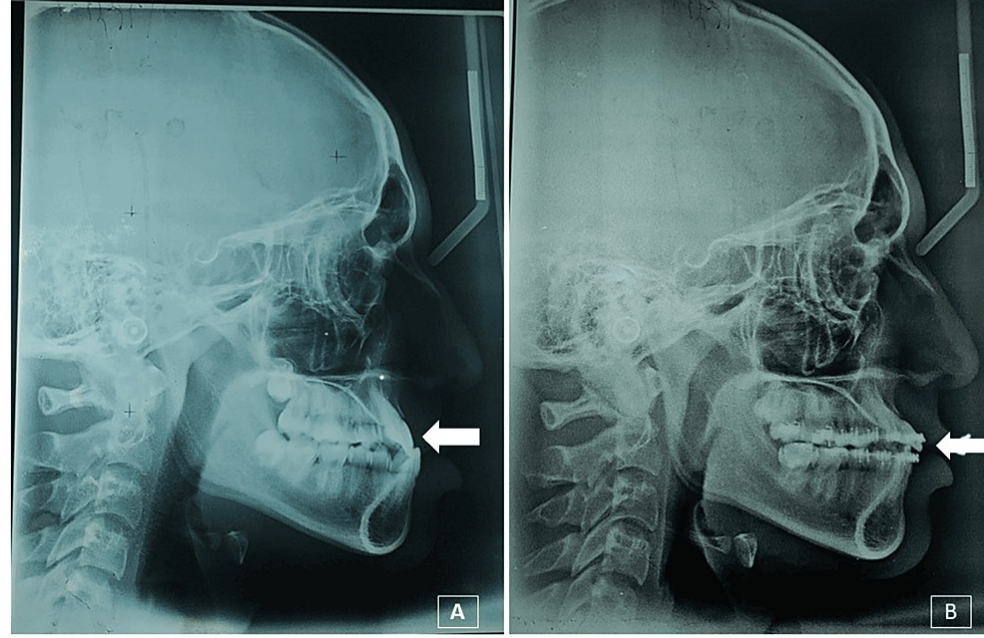
(A) Pre-treatment lateral cephalogram white arrow showing negative overjet. (B) Mid-treatment. Lateral cephalogram with the white arrow showing correction of anterior crossbite

Furthermore, the maxillary arch retained the second deciduous molar, and both right and left premolars exhibited a palatal orientation. The individual displayed a concave facial profile with a deficient midface and a slightly prognathic chin upon inspection. An intraoral examination confirmed a Class III molar and canine relationship on both sides, accompanied by a 2 mm negative overjet of anterior teeth. The upper arch dental midline aligned with the facial midline, while the lower arch dental midline deviated 2 mm to the left. Cephalometric interpretations indicated a Class III jaw relationship attributed to a retrognathic maxilla, an orthognathic mandible, and a horizontal growth pattern (Figure 3).

The patient has overall good health, with no significant abnormalities in their medical history. The treatment objectives aimed to address the discrepancy in arch length, prevent an anterior crossbite, and correct an ectopic maxillary permanent premolar.

Several treatment options were considered for the patient's dental condition. The first option involved extracting the deciduous second molar and repositioning the ectopic premolar to correct its rotation and align it properly. Another option proposed is the removal of the ectopic maxillary premolar, followed by either implant placement or a fixed prosthesis to restore the missing tooth. A third option suggested extracting the ectopic premolar and closing the resulting space by moving the adjacent first molar mesially, which would transition it into a Class II relationship. Each option offered distinct approaches to addressing the ectopic positioning of the premolar, with considerations for alignment, restoration, and overall occlusal harmony.

**Figure 4:**
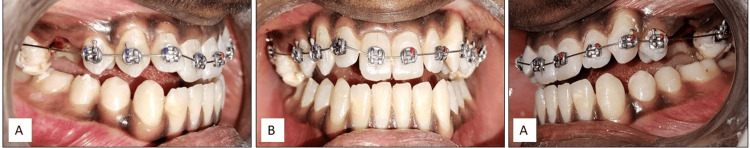
(A) Right and left buccal view respectively, upper arch bonded with bite raised on both sides of the molar. (B) Frontal view showing removal of anterior incisal interferences

The initial treatment option was chosen from all the available options. Following the examination, the patient was recommended to undergo fixed orthodontic treatment. Orthodontic intervention commenced prior to the extraction of the second deciduous molar, utilizing 0.022*0.028 slots (McLaughlin-Bennett-Trevisi MBT PRESCRIPTION) to stabilize both the upper and lower arches. The initial stage of leveling and alignment was successfully accomplished using a 0.016-inch NiTi wire, later followed by the transition to a rectangular wire from a 0.016*0.022-inch Nikle titanium wire. Subsequently, the over-retained primary molar was extracted, and a bite raise was implemented on the lower molar to alleviate crowding, facilitating the proclination of anterior teeth to correct the negative overjet (Figure 4).

**Figure 5:**
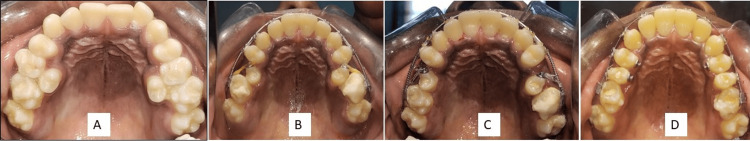
(A) Ectopic eruption of the second premolar with over-retained deciduous second molar. (B) Lingual button attached to pull the premolar. (C) Opencoil given to maintain the space for the second premolar. (D)Alignment of the ectopic premolar

Subsequently, a 0.017*0.025 stainless steel wire was installed in both dental arches. To preserve space between the upper first molar and first premolar, an open coil spring was placed. A lingual button was affixed to the premolar, and targeted forces were applied to rectify its rotation. Following the correction of the rotation, traction forces were applied using a ligature wire. Once the premolar aligned with the arch, a continuous wire was then secured in place (Figure 5).

**Figure 6:**
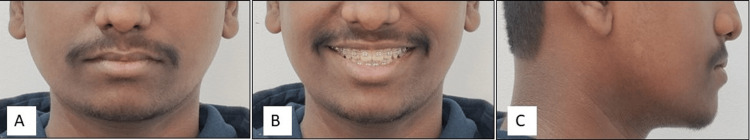
(A) Maxillary arch with ectopic premolar and over-retained second deciduous molar. (B) Mandibular arch. (C) Right occlusal view

Negative torque was applied bilaterally in the root area of the second premolar to achieve alignment. 0.019*0.025 SS wire is placed in the maxillary and mandibular arch with the continuation of Class III elastic. After about 10 months of treatment, the Class I molar and canine relationship was achieved, along with enhancements in the soft tissue profile (Figure 6).

**Figure 7:**
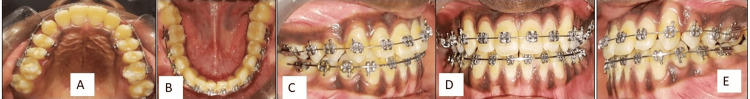
(A) Well-aligned maxillary arch. (B) Mandibular arch. (C) Right buccal view with molar in Class I and canine Class I relation. (D) Frontal view with correction of negative overjet. (E) Left buccal view with molar in Class I and canine Class I relation

Additionally, the ectopic premolar in the maxillary arch is now properly aligned with appropriate intercuspation (Figure 7). The patient's braces haven't been removed yet, as they're still in the stage of final adjustments and refinement.

## Discussion

As parents become more conscious of oral hygiene, there's a growing focus on the ectopic eruption of permanent maxillary premolars at younger ages during the initial phase of intermediate dentition. This condition frequently leads to harm to the roots of the maxillary second primary molars, complicating oral health maintenance [5]. The exact causes of the ectopic eruption of premolars remain unclear, although several factors have been proposed. Genetic predisposition, crowding of the dental arch, abnormal eruption pathways, and disturbances in tooth development are among the potential etiological factors implicated in this condition [6]. Furthermore, environmental factors such as oral habits and dental trauma may also aid in the development of ectopic eruptions. The possibility of being followed from an early stage of development provides numerous opportunities for intercepting and correcting certain types of pathology at the appropriate moment. In this instance, an early intervention enabled the re-establishment of a harmonic dentoskeletal pattern and the laying of the groundwork for proper dentofacial development, as demonstrated by the outcome of this treatment [7].

Xue et al. aimed to establish connections between the ectopic eruption of maxillary premolars and alveolar and maxillary features, offering additional insights for clinical application. Ectopic eruption of premolars in the maxillary arch within the context of Class III malocclusion presents a complex clinical scenario that demands a thorough understanding of the contributing factors, diagnostic considerations, and evidence-based treatment approaches. The intersection of malocclusion and ectopic eruption in Class III patients requires a nuanced approach to achieve optimal outcomes [8]. Early detection and diagnosis of ectopic eruptions are essential for timely intervention and the prevention of further complications. Clinical examination, radiographic evaluation, and orthodontic assessment are integral components of the diagnostic process. Radiographic imaging, including panoramic radiographs and cephalometric analysis, can provide valuable information regarding the position, angulation, and relationship of the ectopically erupted premolar with adjacent structures [9].

The management of ectopic eruptions of premolars often involves a multidisciplinary approach, combining orthodontic, surgical, and restorative interventions. Orthodontic treatment aims to correct malocclusion, align the dental arches, and create adequate space for the ectopically positioned premolar to erupt into its proper place [6]. Surgical procedures such as exposure and orthodontic traction may be necessary to facilitate the eruption of impacted premolars. In severe cases, extraction of the ectopic premolar followed by prosthetic replacement may be considered to restore function and esthetics [10].

The etiology of transposition is not fully understood. It is suspected to have a genetic core within a multifactorial inheritance model, as it is commonly associated with other dental anomalies such as microdontia and impaction. Most transposition cases are likely to be unilateral and are predominantly found in the maxilla. The upper canine transposed with the first premolar and the upper canine transposed with the upper lateral incisor are the most frequently occurring sites. In the mandible, the most commonly occurring transposition involves the canine and the lateral incisor. Management evidence primarily relies on case reports, case series, and opinions. Interceptive treatment in mixed dentition can achieve success; however, its effectiveness is typically limited to scenarios where complete transposition has not yet occurred. Extraction of poorly positioned primary teeth may be considered to facilitate the movement of the permanent tooth into its normal position. Nevertheless, adopting a wait-and-see approach until the child enters the permanent dentition phase might be the most prudent course of action for addressing transposition [11].

Orthodontists must carefully evaluate the effects of ectopic eruption on occlusion, arch symmetry, and periodontal health when planning treatment. Comprehensive orthodontic planning should address not only the correction of malocclusion but also the prevention of potential complications associated with ectopic premolar eruption. Close monitoring of eruption progress, periodic radiographic assessment, and collaboration with other dental specialists are essential aspects of managing ectopic eruptions in orthodontic practice.

## Conclusions

The presented case underscores the complexities involved in addressing dental anomalies such as ectopic premolars and anterior crossbites in adolescent patients. Through a comprehensive treatment approach involving fixed orthodontics, strategic extractions, and meticulous alignment procedures, significant improvements in occlusal harmony and facial aesthetics were achieved. The successful correction of the ectopic premolar alignment and the attainment of a Class I molar and canine relationship demonstrate the efficacy of the chosen treatment modality. This case highlights the importance of tailored orthodontic interventions guided by precise diagnosis and treatment planning to achieve optimal functional and aesthetic outcomes in adolescent patients with complex dental malocclusions.
